# Scientists@Home: What Drives the Quantity and Quality of Online Citizen Science Participation?

**DOI:** 10.1371/journal.pone.0090375

**Published:** 2014-04-01

**Authors:** Oded Nov, Ofer Arazy, David Anderson

**Affiliations:** 1 New York University, New York, New York, United States of America; 2 University of Alberta, Edmonton, Alberta, Canada; 3 University of California, Berkeley, California, United States of America; Bar-Ilan University, Israel

## Abstract

Online citizen science offers a low-cost way to strengthen the infrastructure for scientific research and engage members of the public in science. As the sustainability of online citizen science projects depends on volunteers who contribute their skills, time, and energy, the objective of this study is to investigate effects of motivational factors on the quantity and quality of citizen scientists' contribution. Building on the social movement participation model, findings from a longitudinal empirical study in three different citizen science projects reveal that quantity of contribution is determined by collective motives, norm-oriented motives, reputation, and intrinsic motives. Contribution quality, on the other hand, is positively affected only by collective motives and reputation. We discuss implications for research on the motivation for participation in technology-mediated social participation and for the practice of citizen science.

## Introduction

Recent years have seen a substantial growth in the scale and scope of technology-mediated social participation (TMSP) projects [1], such as Wikipedia, Linux and CiteULike, which rely on volunteers who contribute their time, energy and skills for the creation of a public good [2]. The present study focuses on one type of TMSP: technology-mediated (or ‘online’) citizen science. Like other TMSP efforts, citizen science projects enable members of the public to take part in scientific research addressing real-world problems [3], often through web-based contribution [4]. Various aspects of scientific research - mainly data collection and analysis - are labor-intensive, time-consuming, and consequently costly. Online citizen science reduces the costs of scientific research, increases the resources available to research teams, fosters a partnership between citizens and scientists, and enhances public understanding of science. Scientific breakthroughs such as the discovery of a pulsar by Einstein@home volunteers [5] and the achievements made by contributors to Foldit, an online game in which fold proteins into chemically stable configurations [6], illustrate the potential of this participatory approach to science. Citizen science projects can be characterized by the different levels of task granularity: “the smallest possible individual investment necessary to participate in a project” required from contributors [7]. Task granularities range from the minimal investment required from participants in the case of volunteer computing projects, to more active and demanding tasks in projects that involve distributed data gathering and analysis.

In distributed analysis projects, volunteers engage in image classification or analysis in a variety of scientific areas, which often require task-specific training. Examples include projects like Stardust@home (http://stardustathome.ssl.berkeley.edu) in which volunteers analyze images of interstellar dust particles, and Galaxy Zoo (http://www.galaxyzoo.org), in which volunteers classify images of galaxies [8]. This type of contribution requires some training, and ongoing cognitive effort in analyzing images. A second category of citizen science - distributed data gathering projects - involve geographically distributed inputs from a large number of sources. One example is the Citizen Weather Observer Program (CWOP) in which volunteers provide real-time weather data that is used by research institutions and the weather services. This type of contribution requires offline effort of buying and setting up a weather station, as well as ongoing maintenance of the equipment. Finally, volunteer computing represents the third category, which is based on dividing a large computational task into small tasks that are then distributed over the Internet and completed on volunteers' computers. Well known volunteer computing projects include SETI@home (http://setiathome.ssl.berkeley.edu) and Folding@home (http://folding.stanford.edu). This type of contribution requires the download and installation of a software application, after which contribution is largely passive, and therefore the task granularity is relatively low.

Online citizen science is based on two pillars: (1) a technological pillar, which involves developing computer systems to manage large amounts of distributed resources, and (2) a motivational pillar, which involves attracting and retaining volunteers who would contribute their skills, time, and effort to a scientific cause. While the technological dimension has been widely studied, the motivational dimension of citizen science received little attention to date.

In similar settings, the motivations for contributing to TMSP projects have been studied in variety of projects [9], [10], including Wikipedia [11]–[13], Amazon consumer reviews [14], and open source software (OSS) development [15]–[17]. Nonetheless, it is not clear whether findings from other TMSP settings would carry over to online citizen science settings, as these exhibit some distinctive characteristics that are likely to affect the contributors' motivation. First, in online citizen science there is a clear distinction between the volunteers making the contribution and those benefiting from the aggregate effort (i.e. the scientists who run the project). This asymmetric structure differs from most other TMSP projects (e.g. Wikipedia, YouTube), where the distinction is blurred. Second, the lengthy duration from the time volunteers make their contribution to the time scientific output is made public differs substantially from TMSP projects, where the contributions are viewable immediately, and these differences are likely to influence contributors' motivation. Third, a single contribution to an online citizen science project is sometimes too small to be attributed to a specific individual, whereas in other communities the deliverables (e.g. text, code, or photos) can stand on their own and are usually attributable to their contributor. These differences further stress the need to investigate motivations for participation in the specific context of citizen science [18]. Moreover, results from recent studies of motivation for participation in TMSP demonstrate that the salience of the various motives differ across projects. For example, extrinsic motives (e.g. career advancement, reputation) have been shown to be an important determinant of participation in the case of photo tagging [19], but had no significant effect on the time contributors spent in OSS development [15], and these extrinsic motives had a significant negative effect on engagement in Wikipedia [11]. Thus, it is not clear the extent to which findings from other TPSP projects would carry over to online citizen science.

The objective of this study is, thus, to investigate the motivational drivers of participation in online citizen science projects. We focus our attention on two outcome variables: (i) the quantity of one's contributions (i.e. the number of contributions made in a given period) and (ii) the quality of contributions. With the scant research on TMSP and contribution quality, we expect the comparison of the motivational factors affecting contributions' quantity vs. quantity to yield insights applicable beyond the domain of citizen science.

The paper continues as follows: the next section reviews related work on volunteers' motivations for participation in TMSP, and particularly in citizen science settings; the section that follows develops hypotheses about the relationships between motivations on one hand and contribution quality and quantity on the other; we then describe the research methodology and continue to present the results of our empirical evaluation; the next section discusses our findings, and highlights implications for theory and practice; the final section concludes the paper, pointing to future research directions.

### Background

#### Contributions' Quantity and Quality

The success of TMSP projects depends not only on the amount of users' contribution, but also on its quality [20]. *Quality*, in this context is “a degree of excellence of what is produced”, whereas *quantity* “refers to the total amount of what is produced” ([21] p. 53). While both quantity and quality important, research on the factors underlying TMSP has primarily focused on the quantity of contribution, whereas the drivers of quality have been underexplored, perhaps because the subjective nature of quality and the difficulty to measure it.

Prior applied psychology research has revealed the tradeoff between quantity and quality as a fundamental characteristic of human performance in cognitive tasks [21]–[23]. Although both of these dimensions of human performance are often desirable, due to the limited cognitive resources and information processing capability of an individual they are not always in harmony, as observed in the context of information processing tasks [24] and goal directed behaviors [22], as well as in the context of TMSP (examples include prior studies of discussion forums [20] and online product reviews [14]).

Our study focuses on motivation as an antecedent of both the quantity and quality of contribution to citizen science projects. Empirical evidence suggests that motivational factors may exert differential effects on quantity and quality of contribution in online communities. For example, a study of professional networks of practice found that in the context of an online discussion forum, expectations of reciprocity had a significant effect only on contribution quantity, whereas altruism influenced the quality of contributions [25]. Other studies found that social affiliation, utilitarian motives, and self-expression only affected the quantity of online product reviews submitted, while altruism, reciprocity, and skill development had a significant effect on quality [14]. A related construct - commitment to the community – was shown to have a positive effect on the quantity of photos posted to a photo sharing community, while self-development motives affected the quality of contribution [26]. It is therefore expected that motivational factors exert differential effects on quality and quantity in online citizen science projects.

#### Motivational Factors Affecting Contributions' Quantity

Sustained participation and the sharing of individuals' knowledge are critical for the viability of all online communities [27], [28], and thus an understanding of contributors' motivations for participation [29] is essential for successfully designing and managing TMSP efforts [30], [31]. In recent years, a growing number of studies have investigated volunteers' incentives for sharing information across a wide range of online communities, such as open source projects, Flickr, Twitter, and Wikipedia [1]. Some of the important factors that were found to affect participation include the improvement of skills and enhancement of status [16], [32], enjoyment [19], reciprocity [25], identification with contributors' community [11], [15], and group level factors such as social network properties [33] and group membership size [27].

Within the context of online citizen science, research on motivations for participation is still in an early phase and empirical evidence is scarce. Holohan and Garg [34] studied the motivations for participating in volunteer computing projects and contributing one's computer resources to SETI@home. They found the most salient motivational factors to be the desire to help scientific research, reputation, and gaining technical knowledge, while social factors (being part of team and maintaining social ties) had only secondary importance. A study of motivation for participation in the analysis of galaxy images performed at the Galaxy Zoo project identified ten motivational categories, including: excitement, learning, desire to discover, social interaction, use the project as a resource for [8] teaching, the beauty of the images, fun, amazement by vast scale of the universe, desire to help, interest in the project, interest in astronomy, and interest in science in general. Both these studies provide a descriptive analysis of contributors' motivation, but no attempt is made to link motivations to behavior. A study of SETI@home volunteers [35] on the other hand, developed a research model of the factors determining volunteer computing users' contribution. The results of testing the model exposed personal enhancement and team affiliations to be positively related to computing resource contribution. Overall, however, prior studies have focused on (a) single setting contribution, and (b) contribution quantity rather than quality. What seems to be missing is a broader study of different citizen science settings, focusing on both quantity and quality of contribution.

#### Motivational Factors Affecting Contributions' Quality

A study among authors of Amazon consumer reviews [14] found altruism and reciprocity to be positively related to reviews' quality, whereas a study of Flickr users [26] found the motivation of learning to be positively related to contribution quality. In a more work-related setting, a study of the motivational factors associated with postings in a professional network of practice [25] found that reputation and enjoyment in helping others were positively related with the helpfulness of the postings as perceived by their recipients.

### Theoretical Context

#### Motivation for social participation

Research on participation in social movements has a long tradition in the social sciences [29], [36], [37]. Social movements can be defined from a psychological point of view, as “effort[s] by a large number of people to solve collectively a problem that they feel they have in common” ([38], p. 5). Traditional social movements and technology-mediated social participation are all based on fundamental principle of voluntary contributions made as part of a collective effort, explain why theories of social movement participation have been used in the study of TMSP [11], [15].

A theoretical framework that integrates central findings from social movement research has been developed by Klandermans [39], [40]. According to this framework, the motivation or willingness to participate in a social movement is viewed as a function of the expected costs and benefits from participation. Since the goals of such movements often benefit members of the public regardless of whether they participated in the collective action, the achievement of the goal may be insufficient as a motivating force in and of itself (resulting in social loafing or lurking in online communities). Individuals are therefore likely to have other reasons for participating, which can involve both social and material costs and benefits. Klandermans describes three motives for participation in social movement, with each of the three reflecting a different type of expected cost or benefit: (a) *collective motives* associated with the importance one attributes to the collective goals of the movement; (b) *social motives* resulting from expectations regarding the reactions of important others - such as friends, family or colleagues – to one's participation; and (c) *reward motives* linked to potential benefits to be gained from participation, such as gaining reputation, or making new friends. All three motives are assumed to contribute positively to the willingness to participate in collective action organized by the social movement.

Our study extends the social movement participation model by drawing on Self-Determination Theory [41], in line with recent studies of motivation within TMSP [11]. At the most fundamental level, SDT contrasts extrinsic motivation (in which individuals engage a task in order to achieve a desired outcome) with intrinsic motivation (in which individuals engage in a task out of interest or enjoyment). While Klandermans' discussion of reward motives emphasizes extrinsic rewards [39], [40], intrinsic motives represent an alternative type of reward and were found to play an important role in TMSP [11], [15], [42]. Thus, we include both types of reward motives in our study of citizen science. In order to provide a clear focus, we use in our study one specific type of extrinsic factor, reputation, as this factor has been shown to be an important driver of participation within TMSP in a variety of projects [25], [43], [44]. For clarity reasons, and in line with [15], we refer to the motives related to the expected reactions of important others such as family and friends as *norm-oriented motives*.

In sum, the framework used in our study includes these four motivational factors – *collective motives*, *norm-oriented motives*, *intrinsic rewards*, and *reputation* - as antecedents of both the quantity and quality of participation in online citizen science projects.

#### Hypotheses Development

Collective motives have been found to be important factors in social movements [39] and volunteering [45]. For instance, Simon and colleagues [46] report on two studies of social movements where collective motive had a significant effect on the willingness to participate. In the context of TMSP, however, the effects of collective motives have been less consistent. In Wikipedia, Nov [12] reported statistically insignificant correlation between Wikipedia ideology and participation, and similarly, Schroer and Hertel [11] found the effect of collective motives on engagement to be insignificant. In the context of OSS development, on the other hand, collective motives seem to be more salient. Lakhani and Wolf [16] found that a third of OSS developers studied considered collective motives important. Hertel and colleagues [15] reported that collective motives had a significant effect of on “the willingness to be involved in the future” in OSS contribution. In citizen science, similarly to OSS development, participation is organized around projects, each having a distinct goal, and a collective identity that is derived from the project's goal and ideology. For example, participants in the Stardust@home project refer to themselves as “dusters”. Such high identification with the project's ideology is often a key determining factor in volunteers' decision to join a project, as well as a driver of ongoing participation. In line with the effects observed in open source projects [15], [16], we therefore expect that collective motives in citizen science projects will have positive impact on the quantity of participants' contributions. Formally stated:

*H1a: Higher level of collective motive will be associated with greater contribution quantity.*

Collective motives can also influence the *quality* of contribution. While the notion of ‘quality of contribution' is not directly applicable to social movements (for which the Klandermans model was originally intended), and the antecedents of contribution quality in TMSP have also been underexplored, there is some evidence suggesting that when contributors share the projects' values their contribution to it will be of a higher quality. For example, in OSS development, values associated with the project, such as sharing information and helping others, were found to be related to trust and quality of communication among contributors, which were in turn related to project success [47]. We, thus, expect collective motives to affect quality of contributions in citizen science projects, and we hypothesize:

*H1b: Higher level of collective motive will not be associated with a higher contribution quality.*

Social norms play an important part in enforcing participation in support communities [48] and social movements [39], [49]. In addition, the Information Systems literature shows a positive relation between subjective norms (i.e. the perceived social pressure to engage or not to engage in a behavior) and intended usage behavior [49]. However, empirical evidence of the effects of social norms on volunteering is mixed. For example, Piliavin et al. [50] found that individuals donate blood due to external, social motives, while Simon et al. [46] found in both his studies of participation in social movements that norm-oriented motives had only a marginally significant effect on the ‘willingness to participate’; finally, Houle et al. [51] found that the salience of norm-oriented motives differs between volunteering tasks. Such mixed evidence is also apparent in studies of TMSP. For example, norm-oriented motives did not have a significant effect of the willingness to be involved in the future in OSS projects [15], nor did they have a significant effect on Wikipedia engagement [11].

We believe that the mixed evidence of the effect of norm-oriented motives could be attributed – at least to some extent – to an under-specification of the outcome variable. Perceived social pressure from important others, such as friends and family members, could encourage one to register and contribute to a project from which everyone can benefit, but are less likely induce the kind of commitment, enthusiasm and sustained effort that are necessary for making high-quality contribution. In other words, individuals whose participation is driven primarily by perceived social pressure may not be doing their best as far as contribution quality goes. Such a basic level of participation may be sufficient for an individual to communicate to important others that he is now also a member of their volunteer project. We, thus, expect that norm-oriented motives may affect the *quantity* of contributions made to online citizen science projects, but not the *quality*. Formally stated:

*H2a: Higher level of norm-oriented motives will be associated with a higher contribution quantity.*

*H2b: Higher level of norm-oriented motives will* ***not*** *be associated with a higher quality of contribution.*

Social exchange theory [52] posits that individuals engage in social interaction based on an expectation that it will lead in some way to social rewards such as approval, status, and respect. Specifically, reputation is an important asset that an individual can leverage to achieve and maintain status within a collective [53] and one way in which an individual can benefit from active participation in group activity is through the enhancements of his personal reputation. In corporate settings, results from prior research on electronic networks of practice provide evidence that building reputation is a strong motivator for active participation [54]. In the context of social movements and voluntarism, perceived individual costs and benefits associated with the voluntary engagement are an important driver of participation [39]. For example, Gidron [55] found that young volunteers tended to view their volunteer work as a self-development experience, and Beale [56] suggested that students are interested in volunteering as a stepping stone to employment.

Prior TMSP research demonstrates the enhancement of personal status in the community is associated with the amount of participation in online communities [19], [57], as well as in OSS development [16], [42]. In online citizen science projects, volunteers' level of participation is often measured and presented to the contributor and to other volunteers. These measures commonly take into consideration both the quality and quantity of contribution and are a useful tool for enticing participation. For example, Starust@home displays on the project website a list of the top 100 project volunteers by their score - the number of images classified correctly minus the number of images classified incorrectly, and BOINC provides details of the quantity of computing resources contributed by volunteers over time. We therefore anticipate that:

*H3a: Higher level of reputation will be associated with a higher quantity of contribution.*

*H3b: Higher level of reputation will be associated with a higher quality of contribution.*

Enjoyment has been established as one of the prominent factors explaining volunteering and charitable behavior [58] and is part of the personal benefits to participation in social movements [39]. In the context of online communities, enjoying the act of sharing has been shown to be a prominent reason for contributing to OSS projects ([15], [16], [42], [59], Wikipedia [11], [12] and Amazon online consumer reviews [14]. We, therefore propose:

*Hypothesis 4a: Higher level of intrinsic motivation will be associated with a higher quantity of contribution.*

While the enjoyment associated with contribution is likely to drive volunteers to increase their volume of activity, it is not clear that enjoyment would enhance the quality of output in the context of online citizen science. The tasks performed by volunteers at TMSP projects are often mundane tasks in which quality work requires the investment of additional effort, paying special attention to detail, and satisfying certain requirements of the task that may not be enjoyable. As a consequence, intrinsic motives are unlikely to be associated with enhanced quality. For example, the main task at Stardust@home involves watching images, searching for signs of very small interstellar dust particles. Because of the nature of volunteers' tasks in online citizen science, we expect that intrinsic motivation would not lead to enhanced quality of contribution, and we hypothesize:

*Hypothesis 4b: Higher level of intrinsic motivation will not be associated with a higher quality of contribution.*

Intrinsic and extrinsic motivations are not additive as standard economics assumes; rather, there is a complex relationship between the two, often involving crowding effects [60]–[63]. Crowding effects can be subdivided into a crowding out and a crowding in effect. The crowding out effect involves a negative relationship between intrinsic and extrinsic motivation, and is typical of settings where external incentives are perceived to exert control over members of an organization, such that self-determination is reduced and intrinsic motivation is undermined. The crowding in effect, on the other hand, involves a positive relationship between intrinsic and extrinsic motivation. An outside intervention through rewards or feedback strengthens intrinsic motivation in a trusting environment when members' relationship with the organization are reciprocal and their goals are aligned, such that the intervention is perceived to support intrinsic motivation. Prior studies provide substantial empirical evidence for both types of crowding effects [64], [65].

Crowding-in effects has been reported in the study of how constitutional and legal rules affect citizens [64]. Intrinsic motivation and civic virtue are bolstered when public laws convey the notion that citizens are trusted, such that citizens hold extensive rights, determine their own participation and can organize to influence decision-making processes. Crowding effects have not been studied in the context of citizen science projects. Nonetheless, given that citizen science projects normally assume that volunteer contributors are trusted and that these volunteers are free to determine their participation levels, we can expect a crowding in effect, such that extrinsic motivation (and specifically, reputation motives) would bolster intrinsic motivation. Formally stated:

*Hypothesis 5: Higher level of reputation motivation will be associated with increased level of intrinsic motivation.*

## Methods

### Data collection

We collected data from three different citizen science projects spanning different task granularity levels. Our primary study was conducted at Stardust@home, where we investigated the motivational factors driving both the quantity and quality of contributions. Stardust@home (http://stardustathome.ssl.berkeley.edu/) is a citizen science project based at U.C. Berkeley Space Sciences Lab, in which over 25,000 participants classify images from NASA's Stardust spacecraft, searching for tracks left by very small interstellar dust particles impacting Stardust's aerogel tiles. To enable the identification of the particles, the tiles were scanned, and the scans were divided to small high-resolution images, which were then uploaded to the Stardust@home web-based citizen science system. A “virtual microscope” was developed by the Stardust@home team (see Figure S1) as an interface through which citizen scientists can change the image resolution and point at the location of the suspected tracks. Citizen scientists who join the project undergo a short online training session and then are required to pass a test. After passing their test, the citizen scientists are given online access to the Stardust@home “virtual microscope” and performed the image analysis task. Citizen scientists are awarded positive and negative points for correct and incorrect classifications of tracks and these points are available publicly on the site's leader board.

In addition, we collected data on the antecedents of the quantity of contributions at two other citizen science projects:

The Citizen Weather Observer Program (CWOP) is a weather monitoring citizen science project operated by the National Oceanic and Atmospheric Administration. Volunteers to this project, who installed low-cost weather stations in their homes, record and share weather data online. The data contributed are then made available to various organizations including the US Weather Service, NASA, and a number of research institutions.

The Berkeley Open Infrastructure for Network Computing (BOINC) is a U.C. Berkeley-based platform running volunteer computing projects in various scientific fields. It matches volunteers who contribute computing resources to scientific projects in need of computational power. To participate, volunteers install an application that is then used for managing their computer's allocated tasks. After the initial set-up, contribution is done automatically, with no interaction with the system. Volunteers can set (and change) their level of contribution in a number of ways, for example, by determining the amount of disc space, memory and CPU time allocated to the citizen science project. The BOINC system computes the amount of computer power donated by a participant and allocates computation credit to participants accordingly. BOINC's unit of credit, the Cobblestone is 1/200 day of CPU time on a reference computer that does 1,000 MFLOPS (eventually, credit may reflect network transfer and disk storage as well as computation). Please see http://boinc.berkeley.edu/wiki/Computation_credit for details. Contributors' credit is used as a reputation mechanism: the credit is displayed on both the contributor's computer and the BOINC's web site, motivating participants to contribute more computer resources and consequently build their reputation in the community.

Identical versions of the web-based survey, which differed only in the projects' names, were administered to contributors in all three citizen science projects (see Appendix B).While our focus with respect to sampling was to reach to active project participants differences in the sizes of the target populations, as well as access to volunteers' email addresses required the use of different sampling strategies: A random sample of 4954 BOINC volunteers who were active in the three months prior to the survey launch, were sent an invitation to participate in the study. 513 Stardust@home who were active in the three months prior to the survey launch were sent a similar invitation. At CWOP, where email addresses were not available to the project leadership, an invitation to participate was posted on the project forum by the project leader. Overall, 139 Stardust@home volunteers, 2390 CWOP volunteers and 1843 BOINC volunteers participated in the survey, representing response rates of 27.1%, 22.1% and 37.2% respectively, much in line with the response rates in prior studies in this area [66], [67]. After cleaning of data we were left with 1202 BOINC and 1837 CWOP and 139 Stardust@home valid responses.

### Measurement

A survey was developed based on the Klandermans Model [39] and additional sources [11], [15], [42], [57], [59], relying primarily on prior studies that have adapted the Klandermans Model to TMSP settings [11], [15]. The measurement of collective motives was adapted from [15]. Norm-oriented motives were measured based on [15] The measure for reputation was adapted from [15], [42], [57] Intrinsic motives were measured using the scales from [11], [15], [59]. Contributions' quantity was operationalized as the intention to increase participation, in line with the information systems research tradition of using behavioral intention as a proxy for behavior [68]. The survey items were adjusted to the citizen science context, in consultation with projects' leadership. In addition, the survey included questions about the age, gender, and computer expertise of participants, and these data were used as control in our analysis. Participants were asked to rate the importance of the different motives (collective, norm-oriented, reputation, and intrinsic) on a 1-7 Likert scale. A pilot survey was conducted first, and some items were slightly reworded based on feedback received from participants. The quality of contribution at Stardust@home was operationalized by using the project's internal measure of *Sensitivity*, a measure of how well volunteers correctly identify tracks. Sensitivity is defined as the number of tracks a volunteer correctly identified, divided by the total number of images movies they have searched in which there were tracks. Of the 139 participants who were surveyed, 69 were active throughout the twelve months that followed the survey, and their log data was used in the analysis of contribution quality. As common in studies of online communities in which participation is highly skewed [31], [69], [70], we log-transformed the sensitivity data for the analysis.

### Ethics Statement

The study was approved by the Institutional Review Board of the Polytechnic Institute of New York University.

## Results

### Descriptive statistics

Descriptive statistics are presented in Table 1. On average, across all three projects, collective motives were rated highest (6.26 out of 7), followed by intrinsic motives (5.88). Norm-oriented and reputation motives were found to be of secondary importance (4.56 and 3.64 respectively).

**Table 1:** Descriptive statistics for all three projects.

			Stardust @home		CWOP		BOINC	
Construct	Item	Range	Mean	STD	Mean	STD	Mean	STD
Collective Motives	Col1	[1..7]	6.43	0.87	6.09	0.93	6.21	1.09
	Col2	[1..7]	6.45	0.76	6.13	0.85	6.30	1.02
Norm-Oriented Motives	Nov1	[1..7]	4.78	1.34	5.10	1.20	4.20	1.41
	Nov2	[1..7]	4.87	1.11	4.76	1.15	4.08	1.35
	Nov3	[1..7]	4.75	1.32	4.65	1.14	3.87	1.43
Reputation	Rep1	[1..7]	3.62	1.47	4.11	1.48	2.68	1.62
	Rep2	[1..7]	3.64	1.56	3.73	1.44	2.97	1.72
	Rep3	[1..7]	4.20	1.57	4.47	1.37	3.38	1.76
Intrinsic Motives	Int1	[1..7]	6.00	0.84	5.76	1.06	5.18	1.42
	Int2	[1..7]	6.38	0.62	6.05	0.84	5.89	1.13
Age	Age1		46.32	15.26	52.30	15.56	42.56	14.09
Gender	Gen1	0/1	0.78	0.42	0.97	0.17	0.94	0.24
Expertise	Exp1	[1..7]	5.36	1.11	5.87	0.98	5.56	1.23
Quantity of Contributions	Qnt1	[1..7]	5.33	1.34	4.77	1.24	4.48	1.61
	Qnt2	[1..7]	5.42	1.35	4.63	1.23	4.82	1.61
Quality of Contributions	Qual1	[0..1]	0.67	0.30				

### Measurement Model: Convergent and Discriminant Validity

The convergent validity of our measurement model was assessed in several ways, and repeated for each of the three projects (at Stardust@home, CWOP, and BOINC). First, we examined several competing measurement models to see if they provided a better explanation for our data. This is particularly relevant for the set of self-reported measures, where the possibility of common-method variance introduces an alternate measurement model [71]. Confirmatory factor analyses were then performed using LISREL 8.80 [72]. In total, we compared three different measurement models: (a) the null model (all indicator variables are independent), (b) a one-factor model (all indicator variables load on a single factor), and (c) a six-factor model measurement model: four indicators of motivational constructs, one indicator of contributions' quantity, and one indicator of the control variable (computer expertise). Of the three models tested, clearly, the proposed (six factor) measurement model provided the best explanation for the observed variance and covariance among the set of self-reported indicator variables, and this result was consistent for the three studies at Stardust@home, CWOP, and BOINC.

In addition, we employed *Partial Least Squares* (PLS) using the SmartPLS 2.0 software [73] to assess the reliability of our measures and the structural model. The PLS algorithm estimates path models using composite variables, sometimes called latent variables, from a number of indicator items, sometimes referred to as manifest variables. In this respect, the variance-based PLS path modeling is similar to covariance-based structural equation modeling (SEM) because both algorithms estimate complex relations between several latent variables simultaneously. Nevertheless, a number of conceptual and formal differences make PLS path modeling especially suited for this study. Although both PLS and SEM may suffer when sample size is very small and with non-normally distributed data [74], the PLS algorithm performs better in these conditions and is more robust when assumptions of normality are violated [75], [76]. This was an important consideration for choosing to use PLS in our study, given that some of the variables are not normally distributed.

Using PLS, an index of internal consistency was computed for each multi-item scale. Composite reliability values of 0.89–0.96, 0.89–0.98, and 0.89–0.95 were found at Stardust@home, CWOP, and BOINC respectively (see Tables 2–4). In addition, we analyzed the individual loadings items on their corresponding underlying factor, as well as by the Average Variance Extracted (AVE). All item loadings on their intended constructs were greater than 0.70 and substantially higher than the cross-loadings (see Appendix A). The AVE for each construct was 0.73–0.93, 0.73–0.93, and 0.73–0.91 at Stardust@home, CWOP, and BOINC respectively, substantially greater than the suggested threshold of 0.50 [77]. See Tables 2–4 for details.

**Table 2:** Stardust@home: AVE, Composite Reliability, square-root of AVE (on diagonal; bold) and correlation between the latent constructs.

			Correlations								
Stardust @home	AVE	Comp Rel.	Collective	Norm	Reputation	Intrinsic	Age	Gender	Expertise	Quantity	Quality
Collective Motives	0.88	0.93	**0.94**								
Norm-Oriented Motives	0.73	0.89	−0.09	**0.85**							
Reputation	0.76	0.91	−0.08	0.13	**0.87**						
Intrinsic Motives	0.82	0.90	0.20	0.17	0.18	**0.91**					
[Control] Age	1.00	1.00	0.09	−0.09	−0.22	−0.22	**1.00**				
[Control] Gender	1.00	1.00	0.26	−0.03	0.06	−0.14	−0.01	**1.00**			
[Control] Expertise	1.00	1.00	0.25	0.14	−0.02	0.02	−0.16	0.01	**1.00**		
Quantity of Contributions	0.93	0.96	0.24	0.30	0.14	0.45	−0.33	0.05	0.17	**0.97**	
Quality of Contributions	1.00	1.00	0.38	−0.10	0.07	−0.02	0.05	0.07	0.25	0.07	**1.00**

**Table 3:** CWOP: AVE, Composite Reliability, square-root of AVE (on diagonal; bold) and correlation between the latent constructs.

			Correlations							
CWOP	AVE	Comp Rel.	Collective	Norm	Reputation	Intrinsic	Age	Gender	Expertise	Quantity
Collective Motives	0.83	0.91	**0.91**							
Norm-Oriented Motives	0.73	0.89	0.27	**0.85**						
Reputation	0.79	0.92	0.19	0.46	**0.89**					
Intrinsic Motives	0.81	0.89	0.36	0.51	0.40	**0.90**				
[Control] Age	1.00	1.00	−0.01	−0.08	−0.06	−0.08	**1.00**			
[Control] Gender	1.00	1.00	−0.04	−0.08	0.01	−0.06	−0.01	**1.00**		
[Control] Expertise	1.00	1.00	0.16	0.07	−0.02	0.08	−0.11	0.02	**1.00**	
Quantity of Contributions	0.93	0.96	0.26	0.46	0.58	0.47	−0.13	0.00	0.01	**0.96**

**Table 4:** BOINC: AVE, Composite Reliability, square-root of AVE (on diagonal; bold) and correlation between the latent constructs.

			Correlations							
BOINC	AVE	Comp Rel.	Collective	Norm	Reputation	Intrinsic	Age	Gender	Expertise	Quantity
Collective Motives	0.91	0.95	**0.96**							
Norm-Oriented Motives	0.73	0.89	0.21	**0.85**						
Reputation	0.76	0.90	−0.05	0.28	**0.87**					
Intrinsic Motives	0.79	0.89	0.40	0.34	0.23	**0.89**				
[Control] Age	1.00	1.00	0.03	−0.10	−0.06	−0.07	**1.00**			
[Control] Gender	1.00	1.00	0.01	0.04	0.01	0.05	−0.01	**1.00**		
[Control] Expertise	1.00	1.00	0.04	0.09	−0.02	−0.01	−0.09	−0.16	**1.00**	
Quantity of Contributions	0.90	0.94	0.20	0.33	0.39	0.41	−0.19	−0.06	0.12	**0.95**

We assessed discriminant validity by comparing the square root of the AVE (RAVE) of a particular construct (see Tables 2–4 on the diagonal, in bold) and the correlation between that construct and other latent constructs (presented by the off-diagonal position of the tables). We found that the constructs' RAVE ranges were 0.85–0.97, 0.85–0.96, and 0.85–0.96 at Stardust@home, CWOP, and BOINC respectively, while correlations between constructs were generally below the recommended threshold of 0.5 (with two exceptions: at CWOP the correlations between Reputation and Quantity of Contribution was 0.58 and the correlations between norm-oriented and intrinsic motives was 0.51). In addition, RAVE for every construct is substantially higher than the correlation between that construct and all other constructs. Furthermore, the loadings of all items on their intended construct were higher than on other constructs (see Appendix). Having established reliable and construct valid measures, we tested the study hypotheses by assessing the extent to which the proposed (structural) model fit the observed pattern of variance and covariance among the study measures.

### Hypothesis Testing: Assessing the Fit of the Structural Model

**Figure 1:**
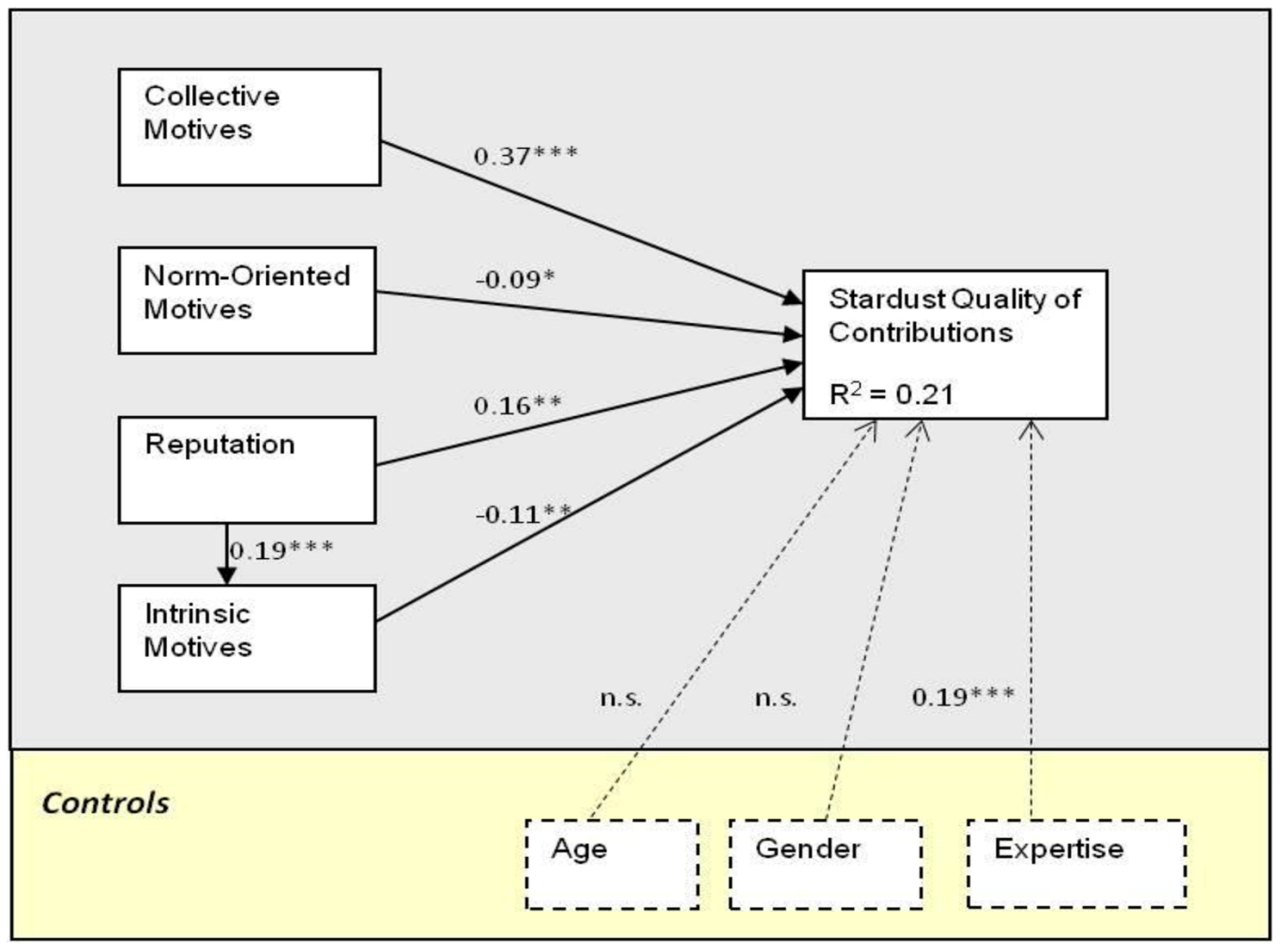
Results of PLS analysis – contribution quantity; Stardust@home/CWOP/BOINC. Please note that ‘*’ p,0.05; ‘**’ p,0.01; and ‘***’ p,0.001.

**Figure 2:**
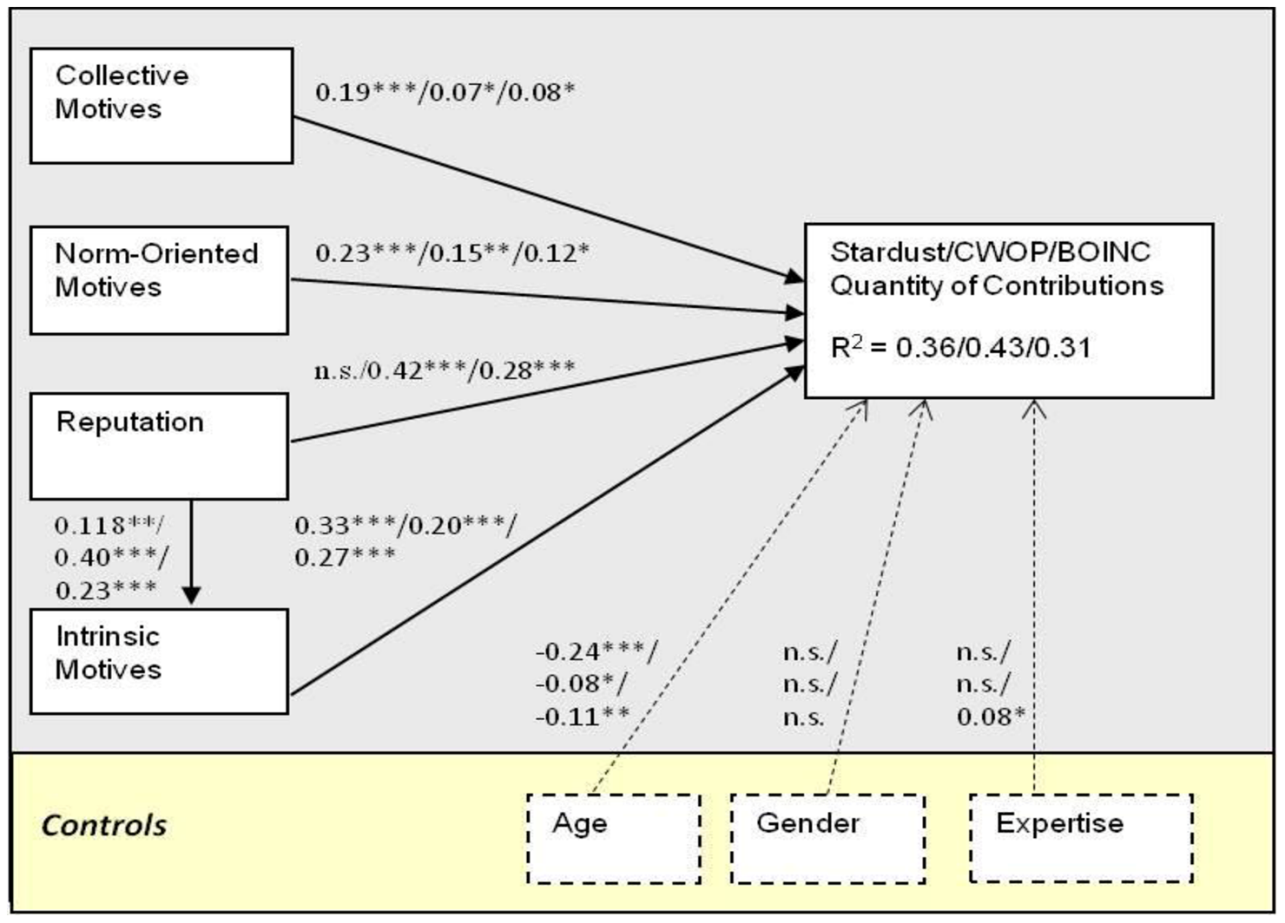
Results of PLS analysis – Stardust@home contribution quality. Please note that ‘*’ p,0.05; ‘**’ p,0.01; and ‘***’ p,0.001.

The specified paths in the structural model corresponded to a large extent to the hypothesized relationships. The significance of structural path estimates was computed using the bootstrapping re-sampling method with 500 re-samples. The structural model was evaluated on the basis of the statistical significance of structural paths and the *R* ^2^ for each composite latent variable. Figure 1 show the results of the PLS analysis for the model explaining contributions' quantity at Stardust@home, CWOP, and BOINC. Figure 2 shows the results of the PLS analysis for the model explaining contributions' quality at Stardust@home, demonstrating a significant positive relations between contribution quality and both reputation and collective motives, and a negative relation with intrinsic motivation. In addition, a positive relation was found between reputation and intrinsic motives.

## Discussion

This study focuses on online citizen science, in which TMSP is channeled to active participation in scientific research projects. The objective of this study was to investigate the relationships between various motivational factors and both the quantity and quality of contribution. Examining three different projects, representing a spectrum of task granularity levels, make the results more generalizable, illustrating the similarities and differences between different types of citizen science projects. The theoretical lens that served the basis for our study was Klandermans' model of participation in social movements [39].

Results from our study show that all four factors – collective motives, norm-oriented motives, reputation, and intrinsic motives – are important drivers of the contribution quantity. Together, the factors analyzed explained 36%, 43%, and 31% of the variance in the outcome variable (for Stardust@home, CWOP, and BOINC respectively). Moreover, these results were consistent across the three different citizen science settings (with one exception discussed below).

While collective motives constitute an important component of Klandermans model, empirical findings from prior TMSP studies yielded mixed results in terms of the impact of these motives on participation. Our findings highlight the importance of these motives across different citizen science settings.

Prior research suggests that norm-oriented motives play an important part in explaining participation in volunteering and social movements [39], [48], and subjective norms are considered an important antecedent of the decision to adopt an information technology [49]. However, empirical studies both offline and online social participation that investigated the effects of social norms on participation have yielded inconclusive results ([46], [51]). The findings of the present study support the notion that while perceived social pressure from important others could encourage volunteers to engage and contribute to citizen science projects, it is less likely to induce the kind of effort and commitment that are necessary for making high-quality contributions.

Individuals often engage in social interaction and collective activity based on an expectation that it will lead in some way to personal rewards, such as enhanced reputation. In line with prior research in TMSP, reputation was shown to be positively related to volunteer participation in the CWOP and BOINC settings.

The fourth antecedent, intrinsic motivation, was found to be positively related to contribution quantity in our three studies, reinforcing our understanding of voluntarism [41] and TMSP [11], [15], [16], [42].

Another finding involves the comparison between the drivers of contribution quantity and quality. By using the same independent variables to study the differences between antecedents of contribution quantity and quality at Stardust@home, we show that while the effect of collective motives is similar for both quantity and quality, there are substantial differences between the effects of the other factors. Norm-oriented and intrinsic motives, which had a positive effect on contribution quantity, did not have a similar effect on quality; in fact, the effects of these factors on the quality of contributions were negative. Norm-oriented motives may play an important role in enforcing participation, but they may be insufficient for driving citizen scientists to exert the effort required for ensuring high-quality outcomes. In a similar manner, the results indicate that enjoyment may entice volunteers to engage with the project, but the additional investment necessary for executing the job well, deem intrinsic motives insufficient for ensuring contribution quality, in line with the findings from online communities [28]. In short, by and large, prior studies of TMSP have focused on the quantity of contribution as the primary outcome (for an exception see [14]). Our study demonstrates that more attention should be given to studying the factors driving contribution quality.

Another interesting result of the comparison of quantity and quality involves the effect of reputation. While at the volunteer computing and weather monitoring settings reputation was found to be an important determinant of participation quantity, at Stardust@home reputation had only an insignificant effect on quantity while having a significant positive effect on contribution quality. A similar effect was observed at Flickr, where extrinsic motives had a positive significant effect on quality, but not on quantity [26]. We believe that the effects of reputation are determined to a large extent by what indicator of performance is made visible publicly. For example, at BOINC the credit of each user is calculated based solely on the quantity of; this information is presented to the user and others in the community. At Stardust@home, on the other hand, a significant emphasis is placed on the *quality* of work.

Our findings from all three settings – across different task granularity levels – reveal a ‘crowding in’ effect, whereby extrinsic motives (i.e. reputation) reinforce intrinsic motivation. The theory suggests that both ‘crowding in’ and ‘crowding out’ (the opposite effect, when external incentives are perceived to be controlling such that self determination is reduced and intrinsic motivation is undermined) are possible, and their manifestation depends on the specific organizational contexts [60]–[63], [78]. Crowding effects have largely not been studied in the context of TMSP. Our findings show that in the three citizen science settings investigated – despite substantial differences the nature of volunteers' tasks and project administration mechanisms – reputation actually acts to enhance volunteers' intrinsic motives. Such an effect is typical of trusting environments that include reciprocal relationship between members and the organization [64], [65], suggesting that citizen science projects have been successful at building a constructive environment where the goals of citizen and professional scientists are aligned.

Finally, the effects of the control variables on the quantity of contribution were highly consistent across the three settings: Age had a significant negative effect, indicating that younger people tend to contribute more frequently; while Gender and Computer Expertise had insignificant effects (except for the significant positive effect of participants' expertise at one setting: Stardust@home). Overall, the controls made a relatively little contribution towards explaining the variance in the quantity of contributions: they added 6%, 1%, and 2% at Stardust, CWOP, and BOINC respectively. When studying the antecedents of contributions’ quality at Stardust@home, Computer Expertise was the only control to have a (positive) significant effect, reflecting the relative complexity of the image analysis task. The three controls have contributed 3% towards explaining the variance in contribution quality.

### Conclusion

The present study contributes to theory in two ways. First, it increases our understanding of what motivates citizen scientists. The second contribution goes beyond the unique citizen science context to the study of TMSP in general, and concerns the differences between contribution quantity and quality in their motivational underpinnings. Our findings from one citizen science projects provide preliminary evidence that factors that enhance participation frequency may not necessarily lead to enhanced contribution quality, and in some case actually detract from quality, calling for a more careful investigation of the effects of motivation.

Information systems research has highlighted the need for contextualizing theory for a specific domain. We argue that given the variety of settings, modalities and content domains in which TMSP is practiced, a context-specific approach is required for TMSP theorizing. Questions of why people participate in social movements can be approached from both macro-level and micro-level perspectives [46]. Thus, an approach that incorporates both micro-level (e.g. motivational) and macro-level (e.g. organizational) factors is needed.

The findings also have important implications for the design and management of online citizen science projects. First, the positive effects of collective motives across projects and outcome variables suggest that citizen science projects should strive to increase volunteers' commitment to the project and its goals. This could be done by communicating the project's mission and achievement to the volunteers. Second, while all motivational factors had a positive effect on the level of participation, the salience of factors differs between projects. Special attention should be therefore given to the unique characteristics of each project setting, and to the effects of these features on motivation. A third implication concerns the factors that determine the quality of contribution. For example, the fact that intrinsic motivation was not found to enhance quality stresses the need to develop more enjoyable, game-like, participation mechanisms, such as the one used in Foldit [6]. Similarly, mechanisms such as social network features should be put in place to create and emphasize social influences, linking them to the quality of one's contributions, so that norm-oriented motives would be positively linked to contribution quality. Finally, motivations could potentially be enhanced by creating a dynamic organization that would allow volunteers to gradually become more involved, and assume responsibility and authority. Many TMSP efforts, such as OSS projects and Wikipedia, have long realized this, and enable volunteers to progress in the tasks they perform and the responsibilities they assume [79]. This mechanism is currently absent in online citizen science projects, where volunteers' tasks are usually restricted in scope, and the governance and decision-making is left in the hands of the professional scientists. Adopting a more nuanced governance structure in which high-performing volunteers are more empowered, would not only enhance citizen scientists' motivations, but may also reduce the load on professional scientists who could delegate some tasks, such as quality control, to volunteers with established track record. Such a change represents a major shift in thinking and theorizing about the relationships between science, scientists and society. However, as online citizen science develops and matures, and demand for volunteers' resource increases, such a trend toward greater volunteer empowerment may be inevitable.

Conclusions drawn from this study should be considered in light of several limitations. First, although using a convenience sample for testing basic psychological mechanisms is often used in information systems research [80], it does limit to some extent the generalizability of the study's findings. Nonetheless, there are no plausible reasons to suggest that volunteers in our sample should differ substantially from the overall population of citizen scientists. Second, there may be some unique features to the three citizen science projects we studied, as there are such features to any online community in general. Future studies of citizen science projects in different fields, with different goals, could help verify the generalizability of the findings. Third, the quality of contributions was empirically studied in only one project and using a relatively small sample, calling for future studies that would repeat the analysis for larger samples and at additional settings. Finally, the study focuses solely on citizen science volunteers; future research may consider the interaction between volunteers and professional scientists.

Online citizen science reduces the costs of scientific research, increases the capacity of research efforts, and fosters a partnership between citizens and scientists. A key challenge to citizen science is the retention of volunteers. The present study advances the understanding of the motivational aspects of online citizen science participation and TMSP in general. Still, a number of key questions warrant future research, including questions concerning the governance of such projects and the design of the online environments supporting participation. We hope that the present study will lead to more research in this field.

## Supporting Information

Figure S1 **A screenshot of the Stardust@home Virtual Microscope.** A volunteer's identification of a track is circled.
(TIFF)

Appendix S1 **Factor loading.** Factor loading.
(DOCX)

Appendix S2 **Survey instrument.** Survey instrument.
(DOCX)

## References

[1] Preece J, Shneiderman B (2009) The Reader-to-Leader Framework: Motivating technology-mediated social participation. AIS Transactions on Human-Computer Interaction 1: 13–32.

[2] Amichai-Hamburger Y (2008) Potential and promise of online volunteering. Computers in Human Behavior 24: 544–562.

[3] Wiggins A, Crowston K. From conservation to crowdsourcing: A typology of citizen science; 2011. IEEE. pp. 1–10.

[4] Hand E (2010) Citizen science: People power. Nature 466: 685–687. 20686547 10.1038/466685a

[5] Knispel B, Allen B, Cordes J, Deneva J, Anderson D, et al (2010) Pulsar Discovery by Global Volunteer Computing. Science 329: 1305. 20705813 10.1126/science.1195253

[6] Cooper S, Khatib F, Treuille A, Barbero J, Lee J, et al (2010) Predicting protein structures with a multiplayer online game. Nature 466: 756–760. 20686574 10.1038/nature09304 PMC2956414

[7] Benkler Y (2006) The wealth of networks: How social production transforms markets and freedom: Yale Univ Press.

[8] Raddick MJ, Bracey G, Gay PL, Lintott CJ, Murray P, et al (2010) Galaxy zoo: Exploring the motivations of citizen science volunteers. Astronomy Education Review 9: 010103.

[9] Wang ES-T, Chen LS-L (2012) Forming relationship commitments to online communities: The role of social motivations. Computers in Human Behavior 28: 570–575.

[10] Amichai-Hamburger Y, Vinitzky G (2010) Social network use and personality. Computers in Human Behavior 26: 1289–1295.

[11] Schroer J, Hertel G (2009) Voluntary engagement in an open web-based encyclopedia: Wikipedians and why they do it. Media Psychology 12: 96–120.

[12] Nov O (2007) What motivates wikipedians? Communications of the ACM 50: 60–64.

[13] Yang H-L, Lai C-Y (2010) Motivations of Wikipedia content contributors. Computers in Human Behavior 26: 1377–1383.

[14] Peddibhotla N, Subramani M (2007) Contributing to public document repositories: A critical mass theory perspective. Organization Studies 28: 327–346.

[15] Hertel G, Niedner S, Herrmann S (2003) Motivation of software developers in Open Source projects: an Internet-based survey of contributors to the Linux kernel. Research Policy 32: 1159–1177.

[16] Lakhani K, Wolf R (2005) Why hackers do what they do: Understanding Motivation Effort in Free. In: J. Feller BF, SHissam , & KLakhani editor. Perspectives in Free and Open-Source Software: MIT Press.

[17] Baytiyeh H, Pfaffman J (2010) Open source software: A community of altruists. Computers in Human Behavior 26: 1345–1354.

[18] Nov O, Arazy O, Anderson D. Dusting for science: motivations and participation of digital citizen science volunteers; 2011; Seattle, WA.

[19] Nov O, Naaman M, Ye C (2010) Analysis of participation in an online photo sharing community: A multi-dimension perspective. Journal of the American Society for Information Science and Technology 61: 555–566.

[20] Gu B, Konana P, Rajagopalan B, Chen HWM (2007) Competition among virtual communities and user valuation: The case of investing-related communities. Information Systems Research 18: 68.

[21] Erez M (1990) Performance quality and work motivation. In: Kleinbeck V, Thierry H, Haecker H, Quast H, editors. Work Motivation: Lawrence Erlbaum Associates. pp. 53–65.

[22] Gilliland SW, Landis RS (1992) Quality and quantity goals in a complex decision task: Strategies and outcomes. Journal of Applied Psychology 77: 672.

[23] Locke EA, Smith KG, Erez M, Chah DO, Schaffer A (1994) The effects of intra-individual goal conflict on performance. Journal of Management 20: 67–91.

[24] Howell WC, Kreidler DL (1963) Information processing under contradictory instructional sets. Journal of Experimental Psychology 65: 39. 13955169 10.1037/h0038982

[25] Wasko M, Faraj S (2005) Why should I share? Examining social capital and knowledge contribution in electronic networks of practice. MIS Quarterly 29: 35–57.

[26] Nov O, Ye C (2009) Why Do People Share Photos Online? Antecedents of Photos' Quality and Quantity. Americas Conference on Information Systems (AMCIS). San Francisco, CA

[27] Butler B (2001) Membership Size, Communication Activity, and Sustainability: A Resource-Based Model of Online Social Structures. Information Systems Research 12: 346–362.

[28] Chiu C-M, Hsu M-H, Wang ETG (2006) Understanding knowledge sharing in virtual communities: An integration of social capital and social cognitive theories. Decision Support Systems 42: 1872–1888.

[29] Snyder M, Omoto A (2000) Doing good for self and society. In: Van Vugt M, Snyder M, Tyler TR, Biel A, editors. Cooperation in modern society: promoting the welfare of communities, states, and organizations: Routledge. pp. 127–141.

[30] Cheshire C, Antin J (2008) The social psychological effects of feedback on the production of Internet information pools. Journal of Computer Mediated Communication 13: 705–727.

[31] Ling K, Beenen G, Ludford P, Wang X, Chang K, et al. (2005) Using Social Psychology to Motivate Contributions to Online Communities. Journal of Computer-Mediated Communication 10..

[32] Oreg S, Nov O (2008) Exploring motivations for contributing to open source initiatives: The roles of contribution context and personal values. Computers in Human Behavior 24: 2055–2073.

[33] Sohn D, Leckenby J (2007) A structural solution to communication dilemmas in a virtual community. Journal of Communication 57: 435–449.

[34] Holohan A, Garg A (2005) Collaboration online: The example of distributed computing. Journal of Computer-Mediated Communication 10: article 16.

[35] Nov O, Anderson D, Arazy O. Volunteer computing: a model of the factors determining contribution to community-based scientific research; 2010. ACM. pp. 741–750.

[36] Della Porta D, Diani M (2006) Social movements: An introduction: Wiley-Blackwell.

[37] Stürmer S, Simon B (2004) The role of collective identification in social movement participation: A panel study in the context of the German gay movement. Personality and Social Psychology Bulletin 30: 263. 15030619 10.1177/0146167203256690

[38] Toch H (1965) The social psychology of social movements. Oxford, England: Bobbs-Merrill Co.

[39] Klandermans B (1997) The social psychology of protest: Blackwell Oxford.

[40] Klandermans B (2003) Collective political action. Oxford handbook of political psychology: 670–709.

[41] Deci E, Ryan R (2000) The "what" and" why" of goal pursuits: Human needs and the self-determination of behavior. Psychological inquiry 11: 227–268.

[42] Roberts J, Hann I, Slaughter S (2006) Understanding the Motivations, Participation, and Performance of Open Source Software Developers: A Longitudinal Study of the Apache Projects. Managenent Science 52: 984–999.

[43] Parameswaran M, Whinston A (2007) Research issues in social computing. Journal of the Association for Information Systems 8: 336–350.

[44] Farzan R, DiMicco J, Millen D, Dugan C, Geyer W, et al.. (2008) Results from deploying a participation incentive mechanism within the enterprise. Proceeding of the twenty-sixth annual SIGCHI conference on Human factors in computing systems. Florence, Italy: ACM. pp. 563–572.

[45] Wilson J (2000) Volunteering. Annual Review of Sociology 26: 215.

[46] Simon B, Loewy M, Stürmer S, Weber U, Freytag P, et al (1998) Collective Identification and Social Movement Participation. Journal of personality and social psychology 74: 646–658.

[47] Stewart KJ, Gosain S (2006) The impact of ideology on effectiveness in open source software development teams. MIS Quarterly 30: 291–314.

[48] Wuthnow R (1993) Acts of compassion: Caring for others and helping ourselves: Princeton Univ Pr.

[49] Pavlou PA, Fygenson M (2006) Understanding and prediction electronic commerce adoption: An extension of the theory of planned behavior. MIS Quarterly 30: 115–143.

[50] Piliavin JA, Evans DE, Callero P (1984) Learning to give to unnamed strangers: The process of commitment to regular blood donation. Development and Maintenance of Prosocial Behavior: International Perspectives on Positive Morality, edited by Ervin Staub, Daniel Bar-Tal, and Janusz Reykowski New York: Plenum Press: 471–492.

[51] Houle BJ, Sagarin BJ, Kaplan MF (2005) A functional approach to volunteerism: Do volunteer motives predict task preference? Basic and applied social psychology 27: 337–344.

[52] Blau PM (1964) Exchange and power in social life: Transaction Publishers.

[53] Jones C, Hesterly WS, Borgatti SP (1997) A general theory of network governance: Exchange conditions and social mechanisms. Academy of Management Review: 911–945.

[54] Kankanhalli A, Tan BCY, Kwok-Kee W (2005) Contributing knowledge to electronic knowledge repositories: An empirical investigation. MIS Quarterly 29: 113–143.

[55] Gidron B (1978) Volunteer work and its rewards. Volunteer Administration 11: 18. 10308779

[56] Beale AV (1984) Exploring careers through volunteerism. School Counselor; School Counselor.

[57] Butler B, Sproull L, Kiesler S, Kraut R (2002) Community effort in online groups: Who does the work and why? Leadership at a Distance: 346–362.

[58] Ribar DC, Wilhelm MO (2002) Altruistic and Joy-of-Giving Motivations in Charitable Behavior. Journal of Political Economy 110: 425–457.

[59] Hars A, Ou S (2002) Working for Free? Motivations for Participating in Open-Source Projects. International Journal of Electronic Commerce 6: 25–39.

[60] Frey B, Oberholzer-Gee F (1997) The cost of price incentives: An empirical analysis of motivation crowding-out. The American economic review 87: 746–755.

[61] Osterloh M, Frey B (2000) Motivation, Knowledge Transfer, and Organizational Forms. Organization Science 11: 538–550.

[62] Osterloh M, Frey B, Frost J (2001) Managing motivation, organization and governance. Journal of Management and Governance 5: 231–239.

[63] Osterloh M, Frost J, Frey B (2002) The dynamics of motivation in new organizational forms. International Journal of the Economics of Business 9: 61–77.

[64] Frey B, Jegen R (2001) Motivation crowding theory. Journal of economic surveys 15: 589–611.

[65] Deci E, Koestner R, Ryan R (1999) A meta-analytic review of experiments examining the effects of extrinsic rewards on intrinsic motivation. Psychological bulletin 125: 627–668. 10589297 10.1037/0033-2909.125.6.627

[66] Wu C, Gerlach J, Young C (2007) An empirical analysis of open source software developers' motivations and continuance intentions. Information & Management 44: 253–262.

[67] Wiggins A, Crowston K. Goals and tasks: Two typologies of citizen science projects; 2012. IEEE. pp. 3426–3435.

[68] Agarwal R, Prasad J (1998) A Conceptual and Operational Definition of Personal Innovativeness in the Domain of Information Technology. Information Systems Research 9: 204–215.

[69] Kittur A, Lee B, Kraut RE (2009) Coordination in collective intelligence: the role of team structure and task interdependence. 27th international conference on Human factors in computing systems. Boston, MA, USA: ACM. pp. 1495–1504.

[70] Burke M, Marlow C, Lento T. Feed me: motivating newcomer contribution in social network sites; 2009; Boston, MA. ACM. pp. 945–954.

[71] Podsakoff PM, MacKenzie SB, Lee JY, Podsakoff NP (2003) Common method biases in behavioral research: a critical review of the literature and recommended remedies. Journal of Applied Psychology 88: 879. 14516251 10.1037/0021-9010.88.5.879

[72] Jöreskog K, Sörbom D (2006) LISREL 8.80. Scientific Software International, Inc.

[73] Ringle CM, Wende S, Will A (2005) SmartPLS 2.0 (beta) Hamburg, Germany.

[74] Qureshi I, Compeau D (2009) Assessing between-group differences in information systems research: A comparison of covariance-and component-based SEM. MIS Quarterly 33: 197.

[75] Chin W (1998) The partial least squares approach to structural equation modeling. In: Marcoulides G, editor. Modern methods for business research: London.

[76] Cassel C, Hackl P, Westlund AH (1999) Robustness of partial least-squares method for estimating latent variable quality structures. Journal of Applied Statistics 26: 435–446.

[77] Fornell C, Larcker D (1981) Evaluating structural equation models with unobservable variables and measurement error. Journal of Marketing Research 18: 39–50.

[78] Frey B (1997) Not just for the money: E. Elgar.

[79] Bryant S, Forte A, Bruckman A (2005) Becoming Wikipedian: transformation of participation in a collaborative online encyclopedia. international ACM SIGGROUP conference on Supporting group work. Sanibel Island, FL: ACM. pp. 1–10.

[80] Ranganathan C, Ganapathy S (2002) Key dimensions of business-to-consumer web sites. Information & Management 39: 457–465.

