# Supplementary figures and images for: Scientists@Home: What Drives the Quantity and Quality of Online Citizen Science Participation?
Source: PLoS One. 2014 Apr 1;9(4):e90375. doi: 10.1371/journal.pone.0090375 (PMC3972171; doi:10.1371/journal.pone.0090375)

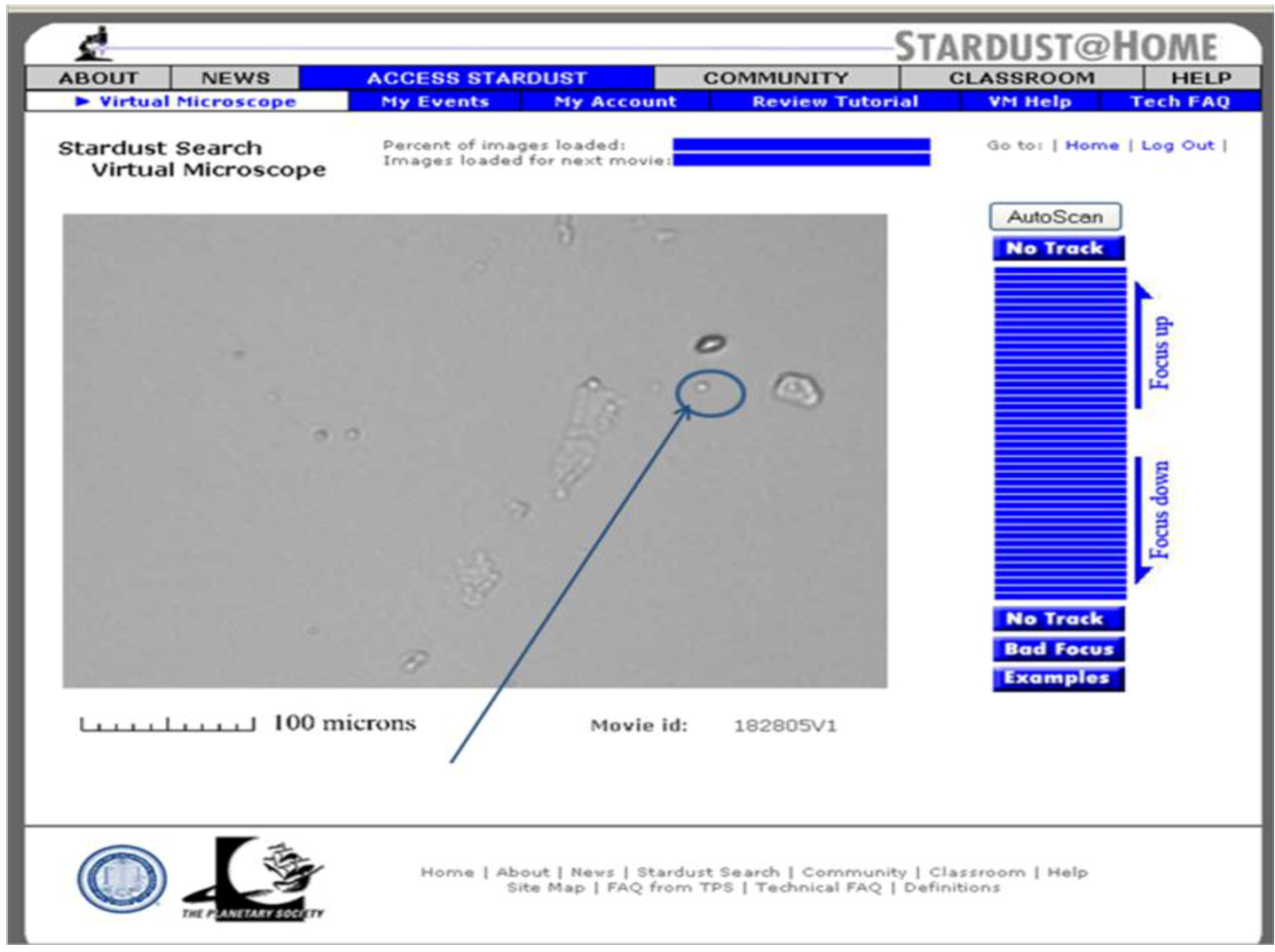

Supplement: Figure S1 — A screenshot of the Stardust@home Virtual Microscope. A volunteer's identification of a track is circled. (TIFF) [file pone.0090375.s001.tiff]
