# Supplementary material for: Scientists@Home: What Drives the Quantity and Quality of Online Citizen Science Participation?
Source: PLoS One. 2014 Apr 1;9(4):e90375. doi: 10.1371/journal.pone.0090375 (PMC3972171; doi:10.1371/journal.pone.0090375)
Supplement: Appendix S1 — Factor loading. Factor loading. (DOCX) [file pone.0090375.s002.docx]

**Appendix S1: factor loading in all projects**

Stardust@home:

| **Stardust** | Collective Motives | Norm-Oriented Motives | Reputation | Intrinsic Motives | Age | Gender | Expertise | Quantity | Quality |
| --- | --- | --- | --- | --- | --- | --- | --- | --- | --- |
| Col1 | **0.95** | -0.14 | -0.02 | 0.19 | 0.06 | 0.27 | 0.19 | 0.23 | 0.39 |
| Col2 | **0.93** | -0.01 | -0.14 | 0.21 | 0.12 | 0.22 | 0.29 | 0.22 | 0.32 |
| Nor1 | -0.13 | **0.85** | 0.03 | 0.10 | -0.12 | 0.05 | 0.04 | 0.31 | -0.09 |
| Nor2 | -0.11 | **0.88** | 0.18 | 0.19 | -0.09 | -0.13 | 0.13 | 0.22 | -0.11 |
| Nor3 | 0.07 | **0.83** | 0.12 | 0.15 | -0.02 | 0.03 | 0.18 | 0.20 | -0.05 |
| Rep1 | -0.05 | 0.15 | **0.92** | 0.14 | -0.26 | 0.06 | -0.04 | 0.24 | -0.01 |
| Rep2 | -0.08 | 0.07 | **0.85** | 0.06 | -0.19 | 0.06 | 0.01 | 0.08 | 0.12 |
| Rep3 | -0.08 | 0.08 | **0.85** | 0.23 | -0.13 | 0.05 | -0.01 | 0.03 | 0.10 |
| Int1 | 0.07 | 0.11 | 0.17 | **0.90** | -0.27 | -0.17 | 0.02 | 0.38 | -0.08 |
| Int2 | 0.30 | 0.18 | 0.16 | **0.92** | -0.12 | -0.08 | 0.01 | 0.44 | 0.04 |
| Age1 | 0.09 | -0.10 | -0.22 | -0.21 | **1.00** | -0.01 | -0.16 | -0.33 | 0.05 |
| Gen1 | 0.26 | -0.01 | 0.06 | -0.13 | -0.01 | **1.00** | 0.01 | 0.05 | 0.07 |
| Exp1 | 0.25 | 0.13 | -0.02 | 0.02 | -0.16 | 0.01 | **1.00** | 0.17 | 0.25 |
| Qnt1 | 0.14 | 0.30 | 0.17 | 0.41 | -0.29 | 0.03 | 0.16 | **0.96** | -0.02 |
| Qnt2 | 0.32 | 0.27 | 0.12 | 0.46 | -0.34 | 0.06 | 0.18 | **0.97** | 0.15 |
| Qua1 | 0.38 | -0.10 | 0.07 | -0.02 | 0.05 | 0.07 | 0.25 | 0.07 | **1.00** |

CWOP:

| **CWOP** | Collective Motives | Norm-Oriented Motives | Reputation | Intrinsic Motives | Age | Gender | Expertise | Quantity |
| --- | --- | --- | --- | --- | --- | --- | --- | --- |
| Col1 | **0.89** | 0.22 | 0.18 | 0.31 | -0.01 | -0.03 | 0.14 | 0.20 |
| Col2 | **0.94** | 0.27 | 0.17 | 0.35 | -0.01 | -0.04 | 0.16 | 0.26 |
| Nor1 | 0.23 | **0.78** | 0.33 | 0.43 | -0.01 | -0.11 | 0.06 | 0.31 |
| Nor2 | 0.24 | **0.89** | 0.40 | 0.46 | -0.07 | -0.05 | 0.05 | 0.38 |
| Nor3 | 0.23 | **0.89** | 0.44 | 0.42 | -0.13 | -0.03 | 0.06 | 0.46 |
| Rep1 | 0.17 | 0.41 | **0.90** | 0.36 | -0.02 | 0.03 | -0.01 | 0.52 |
| Rep2 | 0.15 | 0.40 | **0.91** | 0.32 | -0.04 | 0.00 | -0.02 | 0.52 |
| Rep3 | 0.18 | 0.40 | **0.85** | 0.38 | -0.09 | 0.00 | -0.01 | 0.51 |
| Int1 | 0.30 | 0.47 | 0.39 | **0.90** | -0.08 | -0.06 | 0.07 | 0.41 |
| Int2 | 0.36 | 0.44 | 0.34 | **0.90** | -0.06 | -0.04 | 0.07 | 0.43 |
| Age1 | -0.01 | -0.09 | -0.06 | -0.08 | **1.00** | -0.01 | -0.11 | -0.13 |
| Gen1 | -0.04 | -0.07 | 0.01 | -0.06 | -0.01 | **1.00** | 0.02 | 0.00 |
| Exp1 | 0.16 | 0.06 | -0.02 | 0.08 | -0.11 | 0.02 | **1.00** | 0.01 |
| Qnt1 | 0.25 | 0.44 | 0.56 | 0.44 | -0.13 | 0.00 | 0.00 | **0.96** |
| Qnt2 | 0.24 | 0.44 | 0.56 | 0.45 | -0.12 | 0.01 | 0.01 | **0.96** |

BOINC:

| **BOINC** | Collective Motives | Norm-Oriented Motives | Reputation | Intrinsic Motives | Age | Gender | Expertise | Quantity |
| --- | --- | --- | --- | --- | --- | --- | --- | --- |
| Col1 | **0.94** | 0.20 | -0.05 | 0.37 | 0.03 | 0.01 | 0.02 | 0.16 |
| Col2 | **0.97** | 0.21 | -0.04 | 0.38 | 0.03 | 0.01 | 0.05 | 0.21 |
| Nor1 | 0.18 | **0.78** | 0.13 | 0.30 | 0.01 | 0.06 | 0.01 | 0.23 |
| Nor2 | 0.18 | **0.90** | 0.25 | 0.30 | -0.12 | 0.03 | 0.11 | 0.30 |
| Nor3 | 0.19 | **0.87** | 0.31 | 0.29 | -0.14 | 0.02 | 0.09 | 0.30 |
| Rep1 | -0.06 | 0.23 | **0.87** | 0.17 | -0.07 | -0.08 | 0.03 | 0.33 |
| Rep2 | -0.07 | 0.21 | **0.90** | 0.21 | -0.16 | -0.08 | 0.04 | 0.34 |
| Rep3 | 0.00 | 0.29 | **0.84** | 0.22 | -0.12 | -0.07 | 0.02 | 0.35 |
| Int1 | 0.29 | 0.31 | 0.24 | **0.90** | -0.06 | 0.05 | -0.01 | 0.37 |
| Int2 | 0.43 | 0.31 | 0.17 | **0.88** | -0.06 | 0.03 | 0.00 | 0.36 |
| Age1 | 0.03 | -0.10 | -0.14 | -0.07 | **1.00** | -0.01 | -0.09 | -0.19 |
| Gen1 | 0.01 | 0.04 | -0.09 | 0.05 | -0.01 | **1.00** | -0.16 | -0.06 |
| Exp1 | 0.04 | 0.09 | 0.03 | -0.01 | -0.09 | -0.16 | **1.00** | 0.12 |
| Qnt1 | 0.16 | 0.31 | 0.38 | 0.37 | -0.18 | -0.07 | 0.12 | **0.95** |
| Qnt2 | 0.21 | 0.31 | 0.36 | 0.41 | -0.17 | -0.05 | 0.11 | **0.95** |

**Data availability**

The anonymized data used in this article will be deposited in Dryad and be made available without restrictions.
