# Supplementary material for: Scientists@Home: What Drives the Quantity and Quality of Online Citizen Science Participation?
Source: PLoS One. 2014 Apr 1;9(4):e90375. doi: 10.1371/journal.pone.0090375 (PMC3972171; doi:10.1371/journal.pone.0090375)
Supplement: Appendix S2 — Survey instrument. Survey instrument. (DOCX) [file pone.0090375.s003.docx]

**Appendix S2: Survey Instrument**

**Norm-Oriented Motives**

Nor1: My family thinks positively about my contribution to PROJECT_NAME

Nor2: My friends think positively about my contribution to PROJECT_NAME

Nor3: My colleagues think positively about my contribution to PROJECT_NAME

**Reputation**

Rep1: Gaining reputation in the PROJECT_NAME community is important to me.

Rep2: Having others in PROJECT_NAME appreciate my contribution is important to me.

Rep3: Enhancing my status in the PROJECT_NAME community is important to me.

**Collective motives**

Col1: I value the goals of PROJECT_NAME.

Col2: Overall, advancing the goals of PROJECT_NAME is important to me.

**Intrinsic Motives**

Int1: I enjoy participating in PROJECT_NAME.

Int2: [SPECIFIC PROJECT ACTIVITY] is fun.

**Age**

Age1: [18…99]

**Gender**

Gen1: Female / Male [0 /1]

**Computer expertise**

Exp1: Your level of computer expertise is [Low – High]

**Computer Contributions’ quantity (intention to increase participation)**

Qnt1: I intend to increase my participation in PROJECT_NAME in the future.

Qnt2: My intention is to increase my participation in PROJECT_NAME in the future.
